# High expression of Toll-like receptor 4/myeloid differentiation factor 88 signals correlates with poor prognosis in colorectal cancer

**DOI:** 10.1038/sj.bjc.6605558

**Published:** 2010-02-09

**Authors:** E L Wang, Z R Qian, M Nakasono, T Tanahashi, K Yoshimoto, Y Bando, E Kudo, M Shimada, T Sano

**Affiliations:** 1 Department of Human Pathology, Institute of Health Biosciences, University of Tokushima Graduate School, 3-18-15 Kuramoto-cho, Tokushima 770-8503, Japan; 2 Department of Legal Medicine, Kanazawa Medical University, 1-1 Daigaku, Uchinada, Ishikawa 920-0293, Japan; 3 Department of Medicine, Tsurugi Municipal Handa-Hospital, Tsurugi-cho, Mima-gun, Tokushima 779-4401, Japan; 4 Department of Stress Science, Institute of Health Biosciences, University of Tokushima Graduate School, 3-18-15 Kuramoto-cho, Tokushima 770-8503, Japan; 5 Department of Medical Pharmacology, Institute of Health Biosciences, University of Tokushima Graduate School, 3-18-15 Kuramoto-cho, Tokushima 770-8504, Japan; 6 Division of Pathology, Tokushima University Hospital, 2-50-1 Kuramoto-cho, Tokushima 770-8503, Japan; 7 Department of Surgery, Institute of Health Biosciences, University of Tokushima Graduate School 3-18-15 Kuramoto-cho, Tokushima 770-8503, Japan

**Keywords:** TLR4, MyD88, colorectal cancer, prognosis

## Abstract

**Background:**

The Toll-like receptor (TLR) 4 signalling pathway has been shown to have oncogenic effects *in vitro* and *in vivo*. To demonstrate the role of TLR4 signalling in colon tumourigenesis, we examined the expression of TLR4 and myeloid differentiation factor 88 (MyD88) in colorectal cancer (CRC).

**Methods:**

The expression of TLR4 and MyD88 in 108 CRC samples, 15 adenomas, and 15 normal mucosae was evaluated by immunohistochemistry, and the correlations between their immunoscores and clinicopathological variables, including disease-free survival (DFS) and overall survival (OS), were analysed.

**Results:**

Compared with normal mucosae and adenomas, 20% cancers displayed high expression of TLR4, and 23% cancers showed high expression of MyD88. The high expression of TLR4 and MyD88 was significantly correlated with liver metastasis (*P=*0.0001, *P*=0.0054). In univariate analysis, the high expression of TLR4 was significantly associated with shorter OS (hazard ratio (HR): 2.17; 95% confidence interval (95% CI): 1.15–4.07; *P*=0.015). The high expression of MyD88 expression was significantly associated with poor DFS and OS (HR: 2.33; 95% CI: 1.31–4.13; *P*=0.0038 and HR: 3.03; 95% CI: 1.67–5.48; *P*=0.0002). The high combined expression of TLR4 and MyD88 was also significantly associated with poor DFS and OS (HR: 2.25; 95% CI: 1.27–3.99; *P*=0.0053 and HR: 2.97; 95% CI: 1.64–5.38; *P*=0.0003). Multivariate analysis showed that high expressions of TLR4 (OS: adjusted HR: 1.88; 95% CI: 0.99–3.55; *P*=0.0298) and MyD88 (DFS: adjusted HR: 1.93; 95% CI: 1.01–3.67; *P*=0.0441; OS: adjusted HR: 2.25; 95% CI: 1.17–4.33; *P*=0.0112) were independent prognostic factors of OS. Furthermore, high co-expression of TLR4/MyD88 was strongly associated with both poor DFS and OS.

**Conclusion:**

Our findings suggest that high expression of TLR4 and MyD88 is associated with liver metastasis and is an independent predictor of poor prognosis in patients with CRC.

Colorectal cancer (CRC) is the fourth leading cause of cancer-related death in the world and the third leading cause in the United States (Jemal *et al*, 2009). In Japan, the incidence of CRC has doubled over the past 20 years, and it was the second leading cause of cancer-related death during that period (Tsukuma *et al*, 2004). Although recent advances in chemotherapy have prolonged the survival of patients with advanced disease, the results are still unsatisfactory, and further research is required to understand the disease and improve its outcome (Wolpin *et al*, 2007; Wolpin and Mayer, 2008).

Toll-like receptors (TLRs) expressed on immune cells have a critical role in immune responses against invading pathogens (Akira *et al*, 2006). At least 11 mammalian TLRs have been identified and are involved in recognition by immune and non-immune cells of pathogen-associated molecular patterns, such as lipopolysaccharides (LPSs), viral double-stranded RNA, and unmethylated CpG islands (Akira *et al*, 2006). The TLRs transmit signals through one or more of four adaptor proteins, namely, myeloid differentiation factor 88 (MyD88), MyD88 adaptor-like, Toll/IL1R domain-containing adaptor molecule inducing interferon-*β*, and TRIF-related adaptor molecule (Rakoff-Nahoum and Medzhitov, 2009). Stimulation of TLR leads to the activation of nuclear factor-*κ*B (NF-*κ*B) and mitogen-activated protein kinases, which are essential for the classical outcomes of TLR activation: host innate and adaptive immune responses (Rakoff-Nahoum and Medzhitov, 2009). Toll-like receptor-mediated activation of innate or adaptive immunity may be used as an effective immuno-adjuvant in tumour immunotherapy or combined tumour therapy (Huang *et al*, 2008; Rakoff-Nahoum and Medzhitov, 2009). However, recent evidence has shown that functional TLRs are expressed on a wide variety of tumours (Huang *et al*, 2008; Rakoff-Nahoum and Medzhitov, 2009). Contemporary experimental evidence suggests that TLRs have important roles in tumourigenesis (Killeen *et al*, 2008). Lipopolysaccharide, a putative ligand for TLR4, may promote tumour progression by acting directly on cancer cells, resulting in increased tumour cell–endothelial cell adhesion, tumour cell–extracellular matrix adhesion, and tumour cell–extracellular matrix invasion through NF-*κ*B-mediated upregulation of *β*-1 integrin (Andrews *et al*, 2001; Wang *et al*, 2003). *Helicobacter pylori* acting through TLR2/TLR9 on gastric epithelial cells activated both Src and NF-*κ*B, resulting in an increased expression of cyclooxygenase-2 (Cox-2), which may contribute to gastric cancer progression (Chang *et al*, 2004). The triggering of TLR4 and TLR9 on prostate cancer cells has also been shown to promote tumour cell proliferation through increased NF-*κ*B activation (Kundu *et al*, 2008).

Mounting evidence suggests that innate immune responses to luminal microbes participate in the development of gastrointestinal malignancies (Fukata and Abreu, 2008). The large intestine contains the highest density of microorganisms with the potential to have a key function in colorectal carcinogenesis (Fukata and Abreu, 2008). An expanding body of experimental studies has identified the contribution of TLR signalling to intestinal carcinogenesis (Fukata and Abreu, 2008). Blocking TLR4 signalling in colon cancer cells resulted in the reduction of tumour growth in a subcutaneously implanted mouse model (Huang *et al*, 2005). In addition, the involvement of TLR signalling through MyD88 in tumour growth and progression has been demonstrated in an Apc^min/+^ mice model (Rakoff-Nahoum and Medzhitov, 2007). Furthermore, a recent study reported that TLR4 activation seems to promote the development of colitis-associated cancer by enhancing Cox-2 expression and increasing epidermal growth factor receptor (EGFR) signalling (Fukata *et al*, 2007). These findings have opened a multitude of oncological therapeutic opportunities. However, little is known about the clinical significance of TLR-4/MyD88 signalling expression in sporadic CRC.

In this study, we performed a systematic immunohistochemical analysis of TLR4 and MyD88 expression in normal colon mucosae, adenomas, and CRC. The analysis of the investigation involved two parts: The first was concerned with the correlation between pathological factors and protein expression, and the second tested the correlation between protein expression and disease-free survival (DFS) and overall survival (OS).

## Materials and methods

### Patients and human tissues

A total of 108 patients with CRC (62 males and 46 females) who underwent surgery from 1990 to 1999 at the Tokushima University Hospital were investigated in this study. Information on patient demographics (sex and age) and tumour features (anatomical site, histology, vascular invasion, lymphatic invasion, lymph node metastasis, liver metastasis, peritoneal metastasis, and TNM stage) was obtained from clinical and pathological records (Table 1). The tumour site was classified as proximal or distal with respect to the splenic flexure. Disease stages were classified according to the criteria proposed by the Standard AJCC (American Joint Committee on Cancer) (Fleming *et al*, 1997). Disease-free survival was defined as the interval between the day that surgery was performed and the day that recurrence was first detected. If recurrence was not diagnosed, the date of death or of last follow-up was used. Overall survival was defined as the interval between the dates of surgery and death. The follow-up period after the initial operation for the primary lesion was 5 years for DFS and OS. Exclusion criteria were cancers associated with ulcerative colitis, Crohn's disease, or familial adenomatous polyposis. Ethical approval for the project was obtained from the Tokushima University Hospital Research Ethics Committee.

For the immunohistochemical study, formalin-fixed, paraffin-embedded tissue samples from 15 normal mucosa tissues obtained by colon biopsy, as well as 15 adenomas, 108 CRC, and 14 liver metastases, were used.

### Immunohistochemistry for TLR4 and MyD88

To immunostain TLR4 and MyD88 proteins, the streptavidin–biotin labelling method was carried out on 4 *μ*m tissue sections as described previously (Rahman *et al*, 2009). After deparaffinisation in xylene and rehydration in a series of ethanols, the sections were then treated with 0.3% hydrogen peroxide to block endogenous peroxidase activity for 30 min. Antigen retrieval was performed using an autoclave oven technique. The tissue sections were then incubated with a serum-free protein blocker and incubated at 4°C overnight with mouse monoclonal anti-human TLR4 antibody (1:100; HTA125, eBioscience, San Diego, CA, USA) or rabbit polyclonal anti-MyD88 antibody (1:100; HFL-296, Santa Cruz Biotech, Santa Cruz, CA, USA). The biotinylated secondary antibody and peroxidase-labelled streptavidin (DakoCytomation, Glostrup, Denmark) were applied for 50 min each at room temperature. Antigen–antibody complexes were visualised using the 3, 3′-diaminobenzidine reaction. For confirmation, the 3-amino-9-ethylcarbazole reaction was also used. The slides were counterstained lightly with haematoxylin and mounted for microscopic examination. Human tonsil tissue was used as a positive control for TLR4 and MyD88 immunoreactivity. Phosphate-buffered saline without the primary antibody served as a negative control.

### Evaluation of immunohistochemical findings

Each slide was evaluated independently by two pathologists who were blinded to clinical and outcome data. As most samples stained for TLR4 and MyD88 showed similar colour intensity, from moderate to strong, no evaluation of colour intensity was performed in this study. Any intensity of membrane and/or cytoplasmic staining was considered to represent a positive stain for TLR4 and MyD88. Several high-power fields ( × 200) selected from different staining density regions including high, moderate, low, and negative staining areas were captured using a digital camera (Olympus Q-color 3; Olympus Inc., Center Valley, PA, USA). Photographs were printed on plain paper and a grid was drawn over them. We counted a mean of 2000 tumour cells per tumour (range, 1500–2500), and the results were expressed as the percentage of tumour cells with a positive stain. Thereafter, the percentage of TLR4- or MyD88-positive tumour cells was scored on a scale of 0–4 (0: no staining; 1+: ⩽10% 2+: 11–30% 3+: 31–50% 4+: >50%). Furthermore, the expression levels of TLR4 and MyD88 were divided into the following two groups according to score: low (0, 1+, 2+) and high (3+, 4+). In addition, the sum of the score of TLR4/MyD88 staining was divided into the following two groups: <5 and ⩾5.

### Statistical analysis

Analysis was performed using StatView J-4.5 software (Abacus Concepts, Berkeley, CA, USA). The Pearson *χ*^2^ test or Fisher's exact test was used to compare qualitative variables. The primary statistical outcomes were DES and OS measured from the day of surgery. Both DFS and OS were estimated by Kaplan–Meier curves, and the curves were compared using the log-rank test. Time to relapse and to death was analysed using the Cox proportional hazards model for univariate and multivariate analyses. In addition, the hazard ratios (HRs) between prognostic groups and their 95% confidence intervals were computed. Probability values (*P*) <0.05 were considered to be statistically significant.

## Results

### TLR4 and MyD88 expression in normal colonic mucosae, adenomas, and cancers

**Figure 1:**
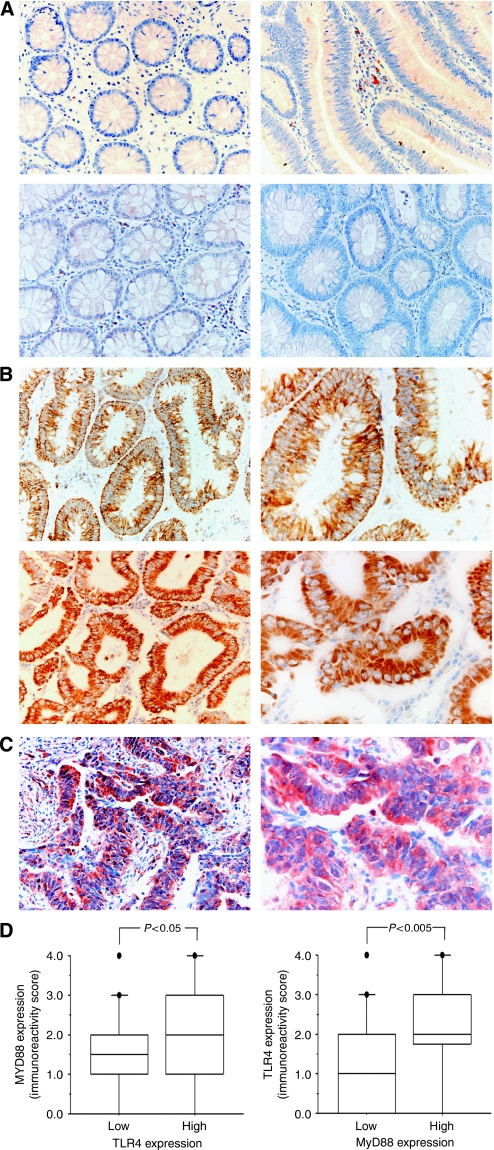
Detection of TLR4 and MyD88 immunoreactivity in normal colon tissues, adenoma, and CRC. (**A**) Normal colon mucosa (left) and adenoma (right) showed no or weak immunoreactions for TLR4 (top) and MyD88 (below), which was identified as negative expression in this study. Original magnification, × 200. (**B**) In contrast to the situation in the normal and adenoma, strong immunostaining of TLR4, which was localized in the membrane (top) or cytoplasm (below), was observed in the cancer cells. Original magnification, right, × 200; left, × 400. (**C**) Strong immunostaining of MyD88, which was localised in the cytoplasm, was observed in the cancer cells. Original magnification, right, × 200; left, × 400. (**D**) High expression of TLR4 was correlated with high expression of MyD88 in CRC. CRC with a high expression of TLR4 (*n*=22) showed significantly high levels of MyD88 expression than did CRC with a low expression of TLR4 (*n*=86) (*P*<0.05). CRC with a high expression of MyD88 (*n*=25) showed significantly high levels of TLR4 expression than did CRC with a low expression of MyD88 (*n*=83) (*P*<0.005).

Immunolocalisation of TLR4 protein was observed in the membrane and cytoplasm. In general, TLR4 expression was absent or very weak in the normal mucosae collected from biopsy samples and cancer margin samples (Figure 1A). This finding is consistent with those of earlier reports (Cario and Podolsky, 2000; Furrie *et al*, 2005; Fukata and Abreu, 2008). The expression of TLR4 was also absent or weak in adenomas (Figure 1A). Compared with normal mucosae and adenomas, the expression of TLR4 was detected in a high proportion of cancers (78 of 108, 72%) (Figure 1B). A total of 22 cancers displayed high expression (4+: 6, 3+: 16), and 86 cancers showed low expression (2+: 33, 1+: 23 and 0: 30) (Table 1). Among the 14 liver metastases obtained by hepatectomy, 12 (86%) were TLR4 positive and 6 (43%) showed a high expression.

Immunolocalisation of MyD88 protein was observed in the cytoplasm. Similar to that of TLR4, MyD88 expression was absent or very weak in normal mucosae, cancer margin samples, and adenomas (Figure 1A). In CRC, a total of 93 of 108 (86%) cancers showed MyD88 expression (Figure 1C). A high-level expression was detected in 25 cancers (23%) (4+: 10, 3+: 15). In all, 83 cancers showed a low-level expression (2+: 33, 1+: 35, and 0: 15). All of 14 liver metastases were positive for MyD88 expression and 12 (86%) of them displayed high expression.

In the co-expression analysis, MyD88 expression levels were higher in TLR4 high-expression CRC than in TLR4 low-expression CRC (*P*<0.05, Figure 1D). Similarly, significantly high levels of TLR4 were detected in CRC with high MyD88 expression (*P*<0.005, Figure 1D). Finally, the correlation between the expression of TLR4 and MyD88 in CRC was confirmed using Pearson's correlation coefficient analysis (*r*=0.33, *P*<0.05) and Spearman's correlation coefficient analysis (*P*=0.003). In addition, 25 (23%) cancers showed a high-level combined expression of TLR4/MyD88 (5+: 12, 6+: 5, 7+: 7, and 8+: 1) (Table 1).

### Clinicopathological significance of TLR4 and MyD88 expression

**Figure 2:**
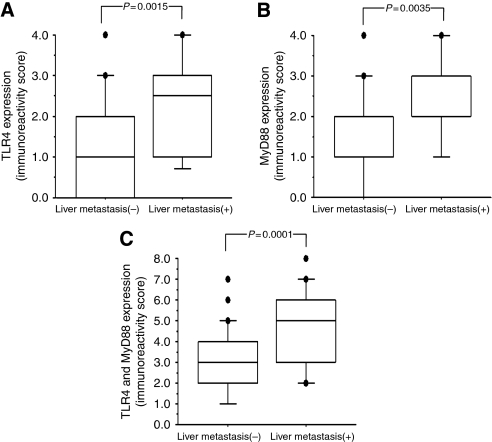
Differences in immunostaining scores of TLR4 and MyD88 in liver metastasis (+) CRC (*n*=22) and liver metastasis (−) CRC (*n*=86) were analysed. The TLR4 (**A**), MyD88 (**B**), and combined TLR4/MyD88 expression (**C**) levels were significantly higher in liver metastasis (+) CRC than in liver metastasis (−) CRC (*P*=0.0015, *P*=0.0035, *P*=0.0001, respectively).

The co-distribution of CRC with a high or low TLR4/MyD88 expression in relation to cancer and patient characteristics is shown in Table 1. The high expression of TLR4 was significantly associated with liver metastasis (*P*=0.0001) and TNM stage (*P*=0.0197) and potentially related to peritoneal metastasis (*P*=0.0611). The high expression of MyD88 was significantly associated with cancer histology (*P*=0.0333) and liver metastasis (*P*=0.0054) and potentially related to TNM stage (*P*=0.0745). Furthermore, a high co-expression of TLR4/MyD88 was significantly associated with vascular invasion (*P*=0.0186), liver metastasis (*P*=0.0002), and TNM stage (*P*=0.0036). In addition, CRC with liver metastasis showed higher levels of TLR4 and MyD88 expression than did CRC without liver metastasis (Figure 2A–C, *P*=0.0015, *P*=0.0035, *P*=0.0001, respectively).

### Clinicopathological parameters and patient survival in CRC

At the 5-year follow-up, 53 patients had tumour recurrence (DFS rate: 49%), and 46 patients had died (OS rate: 43%). In univariate analysis, histology (DFS, *P*=0.0322; OS, *P*=0.0084), TNM stage (DFS, *P*<0.0001; OS, *P*=0.0001), vascular invasion (DFS, *P*<0.0001; OS, *P*=0.0001), lymphatic invasion (DFS, *P*=0.001; OS, *P*=0.0121), lymph node metastasis (DFS, *P*=0.0001; OS, *P*=0.0017), liver metastasis (DFS, *P*<0.0001; OS, *P*<0.0001), and peritoneal metastasis (DFS, *P*=0.0386; OS, *P*=0.007) were important factors associated with DFS and OS (Table 2). Patient age, sex, and tumour location were not related to DFS or OS (Table 2). Multivariate analysis was performed to identify independent prognostic factors. In Table 3, models that include all histopathological variables and tumour markers found to have significant prognostic value in univariate analysis (Table 2) are shown. In Table 3 modelsA and B, TNM stage was significantly associated with DFS (A, *P*=0.0026; B, *P*=0.0043) and OS (A, *P*=0.0074; B, *P*=0.0216), and the presence of liver metastasis was significantly associated with DFS (A, *P*=0.0373; B, *P*=0.047) (Table 3). In Table 3 model B, the presence of lymphatic invasion was significantly associated with DFS (*P*=0.0362), and histology was significantly associated with OS (*P*=0.0435) (Table 3).

### TLR4 and MyD88 expression and patient survival in CRC

**Figure 3:**
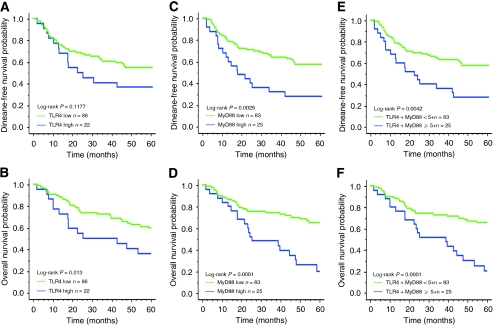
Kaplan–Meier survival curves of DFS and OS in patients with CRC according to TLR4 and MyD88 expression. (**A**, **B**) High expression of TLR4 was potentially associated with poor DFS and significantly associated with poor OS (*P*=0.013). (**C**, **D**) High expression of MyD88 was significantly associated with poor DFS and OS (*P*=0.0029, *P*=0.0001, respectively). (**E**, **F**) High co-expression of TLR4+MyD88 was significantly associated with poor DFS and OS (*P*=0.0042, *P*=0.0001, respectively).

We assessed the influence of high expression of TLR4 and MyD88 in CRC on patient survival. Patients with a high TLR4 expression had a worse DFS than did those with a low TLR4 expression (36 *vs* 55%, Figure 3A). However, a high expression of TLR4 did not significantly influence DFS (log-rank *P*=0.1177; Figure 3A). High expression of MyD88 and high co-expression of TLR4/MyD88 were significantly related to poor DFS (log-rank *P*=0.0029; log-rank *P*=0.0042, respectively, Figure 3C and E). Furthermore, high expression of TLR4, MyD88, and co-expression TLR4/MyD88 were significantly associated with poor OS (log-rank *P*=0.013; log-rank *P*=0.0001; log-rank *P*=0.0001, respectively, Figures 3B, D, and F).

In univariate analysis, high expression of TLR4 was not significantly related to DFS (Table 2). High expression of MyD88 (HR: 2.33; 95% confidence interval (95% CI): 1.31–4.13; *P*=0.0038) and high co-expression of TLR4/MyD88 (HR: 2.25; 95% CI: 1.27–3.99; *P*=0.0053; Table 2) were significantly related to poor DFS. In addition, high expression of TLR4 (HR: 2.17; 95% CI: 1.15–4.07; *P*=0.015), MyD88 (HR: 3.03; 95% CI: 1.67–5.48; *P*=0.0002), and co-expression of TLR4/MyD88 (HR: 2.97; 95% CI: 1.64–5.38; *P*=0.0003; Table 2) significantly influenced OS.

In multivariate analysis of Table 3 model A, high expression of MyD88 was significantly associated with poor DFS (adjusted HR: 2.01; 95% CI: 1.00–4.03; *P*=0.0494) and OS (adjusted HR: 2.31; 95% CI: 1.09–4.89; *P=*0.0279) (Table 3). High co-expression of TLR4/MyD88 was significantly associated with poor OS (adjusted HR: 2.11; 95% CI: 1.05–4.23; *P*=0.0352; Table 3 model B) and potentially related to poor DFS (HR: 1.87; 95% CI: 0.97–3.59; *P*=0.0625; Table 3 model B). As the TNM stage includes metastasis information, we excluded all metastasis factors from Table 4 models and analysed survival significance of TLR4, MyD88, and TLR4/MyD88. In Table 4 model A, multivariate analysis showed that high expression of TLR4 was significantly associated with poor OS (adjusted HR: 1.88; 95% CI: 0.99–3.55; *P*=0.0298; Table 4). In Table 4 model B, high expression of MyD88 was significantly associated with poor DFS and OS (adjusted HR: 1.93; 95% CI: 1.01–3.67; *P*=0.0441; adjusted HR: 2.25; 95% CI: 1.17–4.33; *P*=0.0112, respectively, Table 4). Table 4 model C showed that high co-expression of TLR4/MyD88 was also significantly associated with poor DFS (adjusted HR: 2.06; 95% CI: 1.11–3.82; *P*=0.0216) and OS (adjusted HR: 2.4; 95% CI: 1.28–4.52; *P*=0.0041) (Table 4).

## Discussion

It was recently reported that TLR4/MyD88 signalling drives tumour growth in numerous organs (Rakoff-Nahoum and Medzhitov, 2009). Toll-like receptor 4 and other TLRs have been detected in many murine and human cancer cell lines, including laryngeal, lung, breast, gastric, colon, prostate cancer, and melanoma (Huang *et al*, 2008). Silencing TLR4 signalling in tumour cells results in reduced tumour formation (Huang *et al*, 2005), and the inhibition of tumour-cell apoptosis by TLR-signalling was also observed in ovarian, lung cancer, and myeloma cells (Jego *et al*, 2006; Kelly *et al*, 2006; He *et al*, 2007). In addition, it was shown that MyD88 is crucial for the promotion of diethylnitrosamine-induced hepatocellular tumours (Naugler *et al*, 2007). Recently, MyD88 has also been shown to be crucial for tumour promotion in models of spontaneous (Apc^min/+^) and carcinogen-induced (azoxymethane) intestinal tumourigenesis (Rakoff-Nahoum and Medzhitov, 2007). In addition, MyD88 is a positive regulator of chemically induced tumours of both the skin and connective tissue (Swann *et al*, 2008). In this study, we observed that TLR4/MyD88 signalling was frequently overexpressed in CRC compared with normal mucosae and adenomas. A correlation between TLR4 and MyD88 expression was detected. These findings indicated that TLR4/MyD88 signalling in tumour cells itself has important roles as oncogenic factors in CRC.

It is not yet clear whether TLR4 and MyD88 are involved in tumour initiation (Rakoff-Nahoum and Medzhitov, 2009). In humans, chronic infection and inflammation are considered to be two of the most important factors contributing to tumourigenesis and tumour progression (Balkwill and Coussens, 2004). Toll-like receptor signalling may have an important role in numerous cancers, including gastric, ovarian, lung, pancreatic, liver, and colon cancer, which have been shown to be associated with local chronic inflammation (Kelly *et al*, 2006; Fukata *et al*, 2007; He *et al*, 2007; Naugler *et al*, 2007; Schwartz *et al*, 2009). Upregulated stimulation of TLRs could lead to damage and mutation of genomic DNA and aberrant chromosomal translocation (Rakoff-Nahoum and Medzhitov, 2009). Toll-like receptor 4 signalling activation seems to promote the development of colitis-associated cancer by mechanisms including enhanced Cox-2 expression and increased EGFR signalling (Fukata *et al*, 2007). Excluding colitis-associated cancers, commensal bacteria have also been implicated in the development of sporadic CRC (Hope *et al*, 2005) and may promote CRC by inducing chromosomal instability (Wang and Huycke, 2007). Our findings suggest that TLR4/MyD88 signalling contributes to CRC tumourigenesis not just in colitis-associated cancer but also in sporadic CRC.

The molecular pathway that links inflammation to the acquisition of metastatic capacity during tumour progression has been investigated (Kim *et al*, 2009). By activating TLR2:TLR6 complexes and inducing TNF-*α* secretion in myeloid cells, versican strongly enhances Lewis lung carcinoma metastatic growth (Kim *et al*, 2009). Activation of tumour cell TLR can enhance the invasion and metastasis of tumour cells by regulating metalloproteinases (MMPs) and integrins (Wang *et al*, 2003; Huang *et al*, 2008). The highly invasive MDA-MB-231 breast cancer cell line expresses TLR9, which promotes tumour cell invasion by increasing the activity of MMP-13 (Merrell *et al*, 2006). Toll-like receptor 4 signalling in colon cancer cells is involved in tumour immune escape, accompanied by apoptosis resistance and the preferential induction of immunosuppressive factors and chemokines, such as TGF-*β*, NO, IL-8, MCP-1, and MMP-9, resulting in tumourigenesis and the promotion of tumour metastasis (Cianchi *et al*, 2004; Molteni *et al*, 2006; Wang *et al*, 2003, Lee and Lim, 2007). Earl *et al* (2009) reported that silencing of TLR4 in tumour cells reduces the metastatic tumour burden in steatotic livers. Recently, Killeen *et al* (2009) suggested that bacterial endotoxins directly promote tumour cell adhesion and invasion through upregulation of urokinase plasminogen activator and the urokinase plasminogen activator receptor through TLR-4-dependent activation of NF-*κ*B. Our study showed that TLR4/MyD88 overexpression was frequently detected in CRC with liver metastasis, and TLR4/MyD88 levels were significantly higher in these CRCs. Our findings suggest that TLR4/MyD88 signalling promotes CRC progression by contributing to liver metastasis.

Silencing TLR4/MyD88 signalling in tumour cells not only results in reduced tumour formation but also leads to prolonged survival after subcutaneous tumour injection in mice (Huang *et al*, 2005; Rakoff-Nahoum and Medzhitov, 2007). These effects suggest that blocking TLR4/MyD88 signalling would provide a survival benefit. However, the association between TLR signalling and cancer mortality has not been well investigated in clinical samples. Our results clearly demonstrated that overexpression of TLR4/MyD88 was an independent and significant prognostic factor for 5-year DFS and OS. This is the first study to show an association between TLR4/MyD88 expression and CRC patient survival. Previous studies reported that elevation of the downstream signals of the TLR4/MyD88 pathway, such as Cox-2 and NF-*κ*B, was related to CRC patient survival. We see that TLR4/MyD88 regulates the expression of Cox-2, which shows its important role in many aspects of tumour growth (Fukata and Abreu, 2008; Rakoff-Nahoum and Medzhitov, 2009). Cyclooxygenase-2 is also considered to have an important role in colorectal carcinogenesis. Recently, Ogino *et al* (2008) reported that Cox-2 overexpression is associated with worse survival among colon cancer patients. Nuclear factor-*κ*B is an end point of the TLR4/MyD88 signalling pathway. Numerous lines of evidence that link NF-*κ*B activation to cancer development have been reported. Constitutive NF-*κ*B activation is found in most cancer cell lines and in numerous types of tumour tissues (Shishodia and Aggarwal 2004). Scartozzi *et al* (2007) reported that NF-*κ*B nuclear expression predicts response and survival in irinotecan-refractory metastatic CRC treated with cetuximab–irinotecan therapy. These findings suggested that the tumour cell TLR4/MyD88 signalling pathway has a crucial role in CRC patient prognosis and that blocking this pathway could provide great benefits for patients with CRC.

Recently, several approaches to the treatment of TLR4-mediated diseases have been investigated. Rapamycin may abrogate TLR-triggered colon cancer cell-immune escape and invasion by downregulating TLR4 expression and inhibiting the TLR4-activated NF-*κ*B pathway (Sun *et al*, 2008). Interestingly, TAK-242, a small-molecule antisepsis agent, has been demonstrated to be a selective inhibitor of TLR4 signalling (Kawamoto *et al*, 2008). Our study suggests that it is necessary to investigate the usefulness of TAK-242 in CRC therapy.

Our study profiled the status of TLR4 and MyD88 in CRC and indicated that this signalling pathway was associated with an increased risk of liver metastasis and worse survival. This study, for the first time, showed the clinicopathological significance of tumour cell self-TLR4/MyD88 expression in CRC. These findings suggested the novel therapeutic possibility of targeting tumour cell TLR4/Myd88 signals in CRC.

## Figures and Tables

**Table 1:** Clinicopathological features according to TLR4 and MyD88 expression

	**TLR4**			**MyD88**			**TLR4+MyD88**		
**Clinicopathological factors**	**High (*n*=22)**	**Low (*n*=86)**	***P*-value**	**High (*n*=25)**	**Low (*n*=83)**	***P*-value**	**High (*n*=25)**	**Low (*n*=83)**	***P*-value**
*Age (years)*									
⩾65	10	38	0.915	11	37	0.9593	11	37	0.9593
<65	12	48		14	46		14	46	
									
*Sex*									
Male	14	48	0.5054	13	49	0.534	13	49	0.534
Female	8	38		12	34		12	34	
									
*Tumour site*									
Distal	20	71	0.3113	23	68	0.1972	23	68	0.1972
Proximal	2	15		2	15		2	15	
									
*Histology*									
Well	7	33	0.7685	4	36	**0.0333**	7	33	0.3139
Moderate	13	48		18	43		15	46	
Poor/mucinous	2	5		3	4		3	4	
									
*Vascular invasion*									
Yes	13	34	0.0987	15	32	0.058	16	31	**0.0186**
No	9	42		10	51		9	52	
									
*Lymphatic invasion*									
Yes	18	61	0.3038	20	59	0.3779	18	61	0.8829
No	4	25		5	24		7	22	
									
*Lymph node metastasis*									
Yes	13	51	0.9856	18	46	0.1392	16	48	0.5802
No	9	25		7	37		9	35	
									
*Liver metastasis*									
Yes	11	11	**0.0001**	10	12	**0.0054**	12	10	**0.0002**
No	11	75		15	71		13	73	
									
*Peritoneal metastasis*									
Yes	4	5	0.0611	2	7	0.9452	3	6	0.4666
No	18	81		23	76		22	77	
									
*Stage*									
I, II	6	32	**0.0197**	7	31	0.0745	7	31	**0.0036**
III	5	39		7	35		5	37	
IV	11	17		11	17		13	15	

Abbreviations: TLR4=Toll-like receptor 4; MyD88=myeloid differentiation factor 88. Bold values indicate *P*<0.05.

**Table 2:** Clinicopathological features, tumour markers, and patient survival (univariate analysis)

**Variable**	**5-Year DFS HR (95% CI)**	***P*-value**	**5-Year OS HR (95% CI)**	***P*-value**
Age (⩾65years *vs* <65 years)	0.84 (0.48–1.45)	0.5355	1.17 (0.65–2.08)	0.5934
Sex (male *vs* female)	0.95 (0.55–1.63)	0.8568	0.95 (0.53–1.71)	0.8781
Tumour site (distal *vs* proximal)	2.89 (1.16–8.97)	0.0656	3.17 (0.98–10.23)	0.0534
Histology (poor/moderate *vs* well)	1.95 (1.05–3.6)	**0.0322**	2.57 (1.27–5.19)	**0.0084**
TNM stage (III/IV *vs* I/II)	8.07 (3.2–20.33)	**<0.0001**	6.26 (2.46–15.88)	**0.0001**
Vascular invasion (yes *vs* no)	3.28 (1.86–5.78)	**<0.0001**	3.35 (1.82–6.16)	**0.0001**
Lymphatic invasion (yes *vs* no)	4.68 (1.86–11.79)	**0.001**	3 (1.27–7.09)	**0.0121**
Lymph node metastasis (yes *vs* no)	3.64 (1.87–7.09)	**0.0001**	3.07 (1.52–6.2)	**0.0017**
Liver metastasis (yes *vs* no)	5.04 (2.85–8.9)	**<0.0001**	5.35 (2.94–9.74)	**<0.0001**
Peritoneal metastasis (yes *vs* no)	2.32 (1.05–5.18)	**0.0386**	3.06 (1.35–6.91)	**0.007**
TLR4 (high *vs* low)	1.62 (0.87–2.99)	0.1213	2.17 (1.15–4.07)	**0.015**
MyD88 (high *vs* low)	2.33 (1.31–4.13)	**0.0038**	3.03 (1.67–5.48)	**0.0002**
TLR4+MyD88 (⩾5+ *vs* < 5+)	2.25 (1.27–3.99)	**0.0053**	2.97 (1.64–5.38)	**0.0003**

Abbreviations: DFS=disease-free survival; HR=hazard ratio; CI=confidence interval; OS=overall survival; TNM=tumour, node, metastasis; TLR4=Toll-like receptor 4; MyD88=myeloid differentiation factor 88. Bold values indicate *P*<0.05.

**Table 3:** Clinicopathological features, tumour markers, and patient survival (multivariate analysis)

**Variable**	**5-Year DFS HR (95% CI)**	***P*-value**	**5-Year OS HR (95% CI)**	**P-value**
*Model A*				
Histology (poor/moderate *vs* well)	1.31 (0.67–2.55)	0.4342	1.75 (0.8–3.81)	0.1619
TNM stage (III/IV *vs* I/II)	8.52 (2.11–24.33)	**0.0026**	8.17 (1.76–27.28)	**0.0074**
Vascular invasion (yes *vs* no)	1.32 (0.69–2.54)	0.4034	1.55 (0.77–3.11)	0.2169
Lymphatic invasion (yes *vs* no)	2.53 (0.96–6.67)	**0.061**	1.50 (0.69–3.83)	0.4004
Lymph node metastasis (yes *vs* no)	0.56 (0.21–1.53)	0.259	0.49 (0.15–1.5)	0.2058
Liver metastasis (yes *vs* no)	2.02 (1.04–3.92)	**0.0373**	1.68 (0.79–3.58)	0.1791
Peritoneal metastasis (yes *vs* no)	0.99 (0.43–2.31)	0.9897	1.23 (0.49–3.08)	0.655
TLR4 (high *vs* low)	—	—	1.39 (0.67–2.89)	0.381
MyD88 (high *vs* low)	2.01 (1.00–4.03)	**0.0494**	2.31 (1.09–4.89)	**0.0279**
*Model B*				
Histology (poor/moderate *vs* well)	1.53 (0.81–2.89)	0.1873	2.11 (1.02–4.37)	**0.0435**
TNM stage (III/IV *vs* I/II)	7.17 (1.86–25.68)	**0.0043**	5.56 (1.87–21.68)	**0.0216**
Vascular invasion (yes *vs* no)	1.33 (0.69–2.56)	0.3887	1.35 (0.77–3.11)	0.2224
Lymphatic invasion (yes *vs* no)	2.85 (1.07–7.61)	**0.0362**	1.74 (0.67–4.5)	0.2522
Lymph node metastasis (yes *vs* no)	0.67 (0.25–1.75)	0.4071	0.67 (0.22–1.96)	0.4602
Liver metastasis (yes *vs* no)	1.97 (1.01–3.86)	**0.047**	1.83 (0.87–3.83)	0.1093
Peritoneal metastasis (yes *vs* no)	0.89 (0.38–2.11)	0.8065	1.21 (0.5–2.9)	0.6739
TLR4+MyD88 (⩾5+ *vs* <5+)	1.87 (0.97–3.59)	**0.0625**	2.11 (1.05–4.23)	**0.0352**

Abbreviations: DFS=disease-free survival; HR=hazard ratio; CI=confidence interval; OS=overall survival; TNM=tumour, node, metastasis; TLR4=Toll-like receptor 4; MyD88=myeloid differentiation factor 88. Bold values indicate *P*<0.05.

**Table 4:** Clinicopathological features, tumour markers, and patient survival (multivariate analysis)

**Variable**	**5-Year DFS HR (95% CI)**	***P*-value**	**5-Year OS HR (95% CI)**	***P*-value**
*Model A*				
TLR4 (high *vs* low)	1.31 (0.71–2.43)	0.3816	1.88 (0.99–3.55)	**0.0298**
Histology (poor/moderate *vs* well)	1.82 (0.98–3.37)	0.0572	2.52 (1.24–5.14)	**0.0065**
TNM stage (III/IV *vs* I/II)	5.8 (2.26–15.09)	**0.0003**	4.6 (1.72–12.01)	**0.0013**
Vascular invasion (yes *vs* no)	1.81 (1.01–3.25)	**0.0448**	2.23 (1.18–4.2)	**0.019**
Lymphatic invasion (yes *vs* no)	2.35 (0.91–6.07)	0.0752	1.518 (0.62–3.67)	0.4202
*Model B*				
MyD88 (high *vs* low)	1.93 (1.01–3.67)	**0.0441**	2.25 (1.17–4.33)	**0.0112**
Histology (poor/moderate *vs* well)	1.54 (0.8–2.95)	0.1878	1.89 (0.9–3.99)	0.0679
TNM stage (III/IV *vs* I/II)	5.98 (2.28–15.68)	**0.0003**	4.52 (1.7–11.99)	**0.0017**
Vascular invasion (yes *vs* no)	1.61 (0.87–2.97)	0.122	2.08 (1.09–3.97)	**0.0414**
Lymphatic invasion (yes *vs* no)	2.27 (0.86–5.94)	0.0944	1.38 (0.55–3.45)	0.5922
*Model C*				
TLR4+MyD88 (⩾5+ *vs* < 5+)	2.06 (1.11–3.82)	**0.0216**	2.4 (1.28–4.52)	**0.0041**
Histology (poor/moderate *vs* well)	1.74 (0.93–3.24)	0.0806	2.3 (1.13–4.69)	**0.0155**
TNM stage (III/IV *vs* I/II)	5.89 (2.24–15.45)	**0.0003**	4.48 (1.68–11.92)	**0.0018**
Vascular invasion (yes *vs* no)	1.53 (0.82–2.84)	0.173	1.87 (0.96–3.62)	0.0918
Lymphatic invasion (yes *vs* no)	2.52 (0.97–6.56)	0.0577	1.58 (0.64–3.92)	0.3742

Abbreviations: DFS=disease-free survival; HR=hazard ratio; CI=confidence interval; OS=overall survival; TNM=tumour, node, metastasis; TLR4=Toll-like receptor 4; MyD88=myeloid differentiation factor 88. Bold values indicate *P*<0.05.
